# Diagnostic probability classification in suspected borreliosis by a novel *Borrelia* C6-peptide IgG1- subclass antibody test

**DOI:** 10.3389/fcimb.2023.1108115

**Published:** 2023-09-11

**Authors:** Dag Nyman, Marika Nordberg, Clara Nyberg, Susanne Olausson, Nellie Carlströmer Berthen, Sten-Anders Carlsson

**Affiliations:** ^1^ The Åland Group for Borreliosis Research, Mariehamn, Finland; ^2^ Bimelix Laboratory, Mariehamn, Finland; ^3^ Department of Infection, Åland Public Health Care Services, Mariehamn, Finland

**Keywords:** *Borrelia* infection, probability, IgG1-antibody, serology, diagnostics, C6-peptide

## Abstract

The tick-borne multisystemic infection caused by *Borrelia burgdorferi sensu lato*, Lyme borreliosis, or Lyme disease, occurring in temperate regions of the northern hemisphere, continues to spread geographically with the expanding tick population. Despite the rising perceived risk of infection in the population, the clinical diagnosis of Borrelia infection is not always obvious and the most important laboratory test, antibody detection, has limited accuracy in diagnosing active disease. According to international guidelines, the primary serology test, which has a high sensitivity-low specificity, should, be verified using a high specificity confirmation test to improve the specificity. However, this enhancement in specificity comes at the cost of lower sensitivity. This two-step procedure is often omitted in everyday clinical practice. An optimal primary test would be one where no secondary tests for confirmation would be necessary. In the present study, the performance of a novel assay for quantitating IgG1-subclass antibodies to *Borrelia* C6-peptide was compared to a commercial reference assay of total IgG and IgM antibodies to *Borrelia* C6-peptide in the setting of a high endemic area for borreliosis. A derivation study on a retrospective clinical material was performed to compare the performance parameters and assess the discriminatory properties of the assays, followed by a prospective validation study. The IgG1-antibody assay achieved comparable summary performance parameters to those of the reference assay. The sensitivity was almost 100% while the specificity was about 50%. In a high-endemic setting, characterized by high background seropositivity of about 50% and disease prevalence of approximately 10%, antibody tests are unable to rule-in active *Borrelia* infection. The rule-out assessment of the methods revealed that of 1000 patients, 7 – 54 with negative results based on the reference method could have an active *Borrelia* infection. Such uncertainty was not found for the index test and may help improve the risk classification of patients.

## 1 Introduction

Infection by spirochetes of the *Borrelia burgdorferi sensu lato* (B.b.s.l.) group may cause Lyme borreliosis (LB) or Lyme disease, a multisystemic localized or generalized infectious syndrome with early or late clinical manifestations (Steere, 1989; Stanek and Strle, 2003). The infection is transmitted by hard-bodied ticks and borreliosis is of concern as a common and emerging disease with considerable local epidemiologic variation (Steere et al., 2004).

Despite increasing knowledge of the infectious agent, its transmission, clinical and laboratory diagnosis, and the proven treatment of borrelia infection, there is a growing concern regarding potential undiagnosable *Borrelia* infection and possible chronic forms as well as post-treatment Lyme disease syndrome (PTLDS) (Steere, 2020; Strle and Strle, 2020; Wormser et al., 2020; Ursinus et al., 2021; Geebelen et al., 2022).

The diagnostic process starts with the evaluation of symptoms and signs, giving a pre-test estimate of the probability of the disease.

Infection by *Borrelia* may be asymptomatic with an isolated immune response in 40 – 50%, (Wilhelmsson et al., 2016).

The early skin manifestation, Erythema migrans (EM), is observed in ≥ 80% of LB patients (Steere et al., 2016; Stanek and Strle, 2018) and may attain a clinical pre-test probability of 80% for LB in an endemic region, necessitating immediate antibiotic treatment without further laboratory investigations (Tugwell et al., 1997). Untreated EM may evolve into a disseminated or chronic state in 25 – 40% of the cases (Stanek and Strle, 2018).

Apart from EM, the symptoms and system-specific signs of early and late, disseminated or local *Borrelia* infection are diagnostically unspecific as outlined in European clinical case defining guidelines (Stanek and Strle, 2018), needing further laboratory confirmation.

Early neuroborreliosis (NB) is the main disseminated form occurring in 50 - 60% of cases (Stanek and Strle, 2018), characterized by isolated facial palsy or involvement of other cranial or peripheral nerves and/or subacute meningitis, eventually associated with painful radiculoneuritis. Other local manifestations are borrelia arthritis (LA), borrelial lymphocytoma, and Acrodermatitis atrophicans (ACA), in 10 - 15%, 1 - 2%, and 5 - 10% of cases respectively. Cardiac or ocular manifestations are scarce.

The most important supportive objective investigation at the beginning of the diagnostic work-up is serology. However, the interpretation of the serologic results may be complicated due to inconsistencies in methods and interpretation of the results, which may contribute to underdiagnosis, overdiagnosis, and erratic treatments (Leeflang et al., 2016).

As antibodies are indirect evidence of infectious disease burden, not of active disease, the performance of assays to rule-in active borreliosis is generally poor.

Antibody tests may be negative in active infections with short symptomatic intervals (Raffetin et al., 2022).

We hypothesized that a single, specific peptide-based antibody assay would improve the performance of antibodies as diagnostic support in the LB clinic. The well-characterized borrelial VlsE-related C6-peptide is a major conserved, strongly immunogenic, and infection-related sequence in Borrelia burgdorferi species (Liang and Philipp, 1999; Liang and Philipp, 2000; Embers et al., 2007). Antibodies to the C6-peptide have also been extensively used in clinical diagnostics alone or in combination with a second enzyme-linked immunosorbent assay (ELISA) with reliable results (Jansson et al., 2005; Nyman et al., 2006; Lager et al., 2019; Branda and Steere, 2021). An IgG-antibody would be preferable, excluding IgM-serology. IgM-serology is associated with frequent persistence of false positive IgM, which results in low specificity, necessitating verification by immunoblot (IB) and demonstration of seroconversion to IgG-antibodies (Kalish et al., 2001; Wilske, 2003; Wilske et al., 2007).

IgG1 is the IgG-subclass most reactive in active infections and is less related to the presence of longstanding borrelia seropositivity than antibodies from subclasses 2 and 3 (Olsson et al., 1987; Artsob and Hubner, 1990; Seppälä et al., 1994; Panelius et al., 1999).

An indirect ELISA for determining IgG1 antibodies to borrelia C6-peptide has been developed. In the present work, we describe the performance of this novel antibody test as an aid to clinical probability stratification in the diagnostic evaluation of suspected disseminated or late local LB after a symptomatic phase of at least 2 – 4 weeks.

In the present study, we describe the development and evaluation of a novel test for IgG1-subclass antibodies to B.b.sl. in a hyperendemic European area. The findings demonstrated a low positive predictive performance, placing special emphasis on the rule-out performance of the test.

## 2 Materials and methods

### 2.1 Population, seroprevalence, and prevalence of borrelia infection in the region

The Åland Islands had a population of 30 000 inhabitants in 2021, and an estimated annual incidence rate of generalized or long-standing local *Borrelia* infections, excluding EM, per 100 000 population of 1468, 2136, and 1710 in 2000, 2012, and 2021, respectively. These figures are based on annual laboratory reporting of seropositive samples to the Finnish Institute for Health and Welfare, representing an epidemiologic estimate of the trend of borreliosis over time. The figures represent the sum of definite and probable, disseminated, symptomatic, and asymptomatic infections as well as the seropositive background (Finnish Institute of Health and Welfare, website, 2022).

A study on tick-transmitted infections (Wilhelmsson et al., 2016) of 575 participants from the Åland Islands showed that 49% were seropositive using the reference method described below. Of the participants, 159 (27.7%) were bitten by a B.b.s.l. carrying tick, 3.5% of whom showed seroconversion while 2.6% had clinical *Borrelia* infection. The total infection risk of a single tick-bite causing LB in the participants, calculated from these data was 0.061 (95% CI 0.042 – 0.081).

### 2.2 Retrospective seroprevalence and diagnosis of NB in general patients

During three years, 2017 – 2019, 7185 individual serum samples were sent to the laboratory from local healthcare physicians. The figures comprise the total amount of B.b.s.l.-antibody tests performed in three years. Antibodies to B.b.s.l. were demonstrated in 4286 (59.7%) using the reference method described below. Clinical symptoms suggestive of neuroborreliosis were present in 414 patients (5.7%), and cerebrospinal fluid (Csf) was obtained in all. A diagnosis of NB was made in 44 patients, resulting in a prevalence of 0.6% in patients tested for antibodies to *Borrelia*, a prevalence of 1% in seropositive patients, and a prevalence of 10.6% in those with symptoms of possible NB.

### 2.3 Seroprevalence in healthy blood donors

To assess the actual seroprevalence in healthy persons, serum samples from 312 HBD, 20-69 years of age, were obtained at the Finnish Red Cross blood donation occasion in the Åland Islands in Dec. 2018. There were 175 (56.1%) females and 137 (43.9%) males. Antibodies to B.b.s.l. were measured by the reference method and by the index method.

### 2.4 Antibody assays

The reference method for the assay of serum antibodies to borrelia was the C6 Lyme ELISA Kit™ (Immunetics, USA). In this test, the sums of reactive IgG and IgM antibodies were determined simultaneously. The results were reported in Lyme Index (LI) calculated as (Patient OD value): (Calibrator OD value + 0.3) according to the manufacturer and interpreted as positive if ≥1.1 LI and negative if ≤ 1.09 LI, the indeterminate interval 0.91-1.09 LI was counted as negative. The samples were diluted to obtain the final antibody level.

The results of individual tests were related to the duration of symptoms. A negative antibody test after a symptomatic period shorter than 2 - 4 weeks required a second sample to be taken after 2 - 4 weeks.

An in-house indirect enzyme-linked immunosorbent assay (ELISA) for serum IgG1-antibodies to synthetic borrelia C6-peptide, the index test, was developed in the laboratory.

Synthetic C6-peptide, with the sequence CMKKDDQIAAAMVLRGMAKDGQFALK (Liang et al., 1999) and N-terminally biotinylated, was obtained from Gen-Script USA Inc, Piscataway, NJ, USA, diluted as prescribed by the manufacturer and used for coating of Pierce streptavidin-coated high-capacity plates (Fisher Scientific Oy, Vantaa, Finland), according to the manufacturer’s instructions.

The serum samples were primarily diluted 1:20 with tris buffered saline (25mM Tris, 150mM NaCl; pH 7.2, Thermo Scientific Oy, Product No. 28376), with 0.1% BSA, 0.05% Tween^®^- 20 Detergent, and 100 µl of serum dilutions alternatively buffer, was added to the wells and incubated with shaking at room temperature for 30 min.

After washing with buffer three times, 100 µl of 1:500 dilution in a buffer of Mouse Anti-human IgG1 horseradish peroxidase conjugate (Fisher Scientific Oy) was added to each well and incubation at room temperature with shaking for 30 min. After washing three times, 100 µl tetramethylbenzidine substrate (Thermo Scientific TMB Substrate Kit, Product No. 34021) was added to each well. After incubation in the dark for 30 min. without shaking, 100 µl stop solution was added and the absorbance was read at 450 nm against reference 620 nm.

In order to determine the negative/positive cutoff point, the level of blank (LOB) was measured and the levels of detection (LOD) and quantitation (LOQ) of the test were estimated from the optical density (OD) of LOB (Armbruster and Pry, 2008). The primary LOQ was calculated as LOB + 10 * standard deviation (SD). The working LOQ was determined when the measurement error of LOQ was accounted for by adding the 99.9% upper margin of error, calculated as 3.291 * standard error of the mean (SEM) of ODs at the level of the primary LOQ, to the primary LOQ estimate.

Subsequently, ODs were converted into arbitrary antibody units (AU) by dividing the sample absorbance by 3 * mean absorbance of zero-samples (blank).

### 2.5 Assessment of the clinical cutoff point

The clinical positive/negative reference test cutoff level of 1.1 LI corresponded to 2.22 AU in the index test, calculated from the regression equation obtained in the derivation cohort: (log(y) = 0,3058 + 0,9997log(x)).

The upper limit of the negative interval was calculated by the robust method (CLSI C28-A3) for all results below 2.0 LI (273) for the reference test and below 4.0 AU (287) for the index test. The mean upper limits (90% CI) were 0.928 (0.83-1.04) for the reference test and 1.87 AU (1.64-2.09) for the index test.

Accordingly, values ≥ 2.1 AU were scored positive, and ≤ 2.09 AU were negative. An intermediate, equivocal level was not applied. The samples were diluted to obtain the final antibody concentration.

### 2.6 Derivation cohort, method qualification

A retrospective derivation cohort was used for estimating the technical and diagnostic performances of the index method, compared to the reference method. The cohort size was 431, consisting of 119 defined serum samples from the ScandTick biobank (Lager et al., 2019), including 48 samples from patients with known, clinically confirmed, disseminated or late LB characterized as 41 NB, 3 LA, 2 ACA, 2 LA with ACA. Additionally, 51 non-LB patients with other causes of symptoms and 20 samples from healthy blood donors. The prevalence of LB was 37.8%.

In order to adjust the LB prevalence in the material to represent the local clinical pattern, the data from the 312 local healthy blood donors were added to the ScandTick data. The prevalence of LB in the derivation cohort was then 11.1%.

### 2.7 Clinical validation cohort

A prospective validation cohort of 208 consecutive clinical samples from patients sent to the laboratory by local healthcare physicians from December 2019 to March 2020, was used for validation of the index ELISA diagnostic performance. The laboratory assays by the index test were performed blindly, without knowledge of the results of the reference serology, nor of the final diagnosis.

The sample size was calculated assuming a 15% difference in proportions of negative likelihood ratios (LR-) between the methods with power >0.85 and p<0.01.

The clinical evaluation of referred patients was made by experienced physicians according to European diagnostic guidelines (Stanek and Strle, 2018). An additional diagnostic group designed “Early disseminated LB”, initially called encephalopathy (Halperin et al., 1991), characterized by constitutive symptoms and cognitive impairment for over two weeks after a tick bite, without evident EM or other manifestations of *Borrelia* infection, in combination with seroconversion or rising level of IgG antibodies to borrelia and normal findings in Csf.

In suspected NB general markers of inflammation and organ damage, including albumin, immunoglobulin G, and antibodies to B.b.s.l. were analyzed in serum (S). In Csf total protein, albumin, immunoglobulin G, cells with mononuclear/polynuclear counts, as well as automatic calculation (Reiber and Lange, 1991) of intrathecal specific IgG antibody synthesis (recomBead, Mikrogen, Germany), and Csf-CXCL13 (Rupprecht et al., 2018) chemokine (Euroimmun, Germany) were analyzed.

In clinical arthritis with hydrops, investigations consisted of serum antibodies to B.b.s.l. and markers of inflammation, synovial fluid poly-/mononuclear cell count, protein, crystals, bacteria as indicated, and synovial fluid borrelia-PCR in all samples of synovial fluid. Further investigations were made as required for differential diagnosis.

In suspected ACA serum antibodies to B.b.s.l. and skin biopsy for histology and borrelia PCR of the biopsy were performed.

Differential diagnostic investigations, such as brain MRI, autoimmune panels, and multiplexed pathogen PCR, were made when indicated. Antibiotic treatment with amoxicillin, doxycycline, or ceftriaxone for 21 days was administered based on the clinicians’ judgments. The final diagnosis for the study was determined after a follow-up of three months.

### 2.8 Statistics

Proportions and percentages (%) were reported as mean with a 95% confidence interval (CI). Quantitative antibody levels are summarized as median and range. The significance of the difference between groups was tested by the McNemar test, and proportions were compared by chi-squared test and z-statistic. The Kruskal - Wallis test was used for the evaluation of the distribution of antibody levels between non-LB and LB samples. Differences with p<0.05 were considered significant.

The diagnostic parameters were evaluated using binary diagnostic in a 2 x 2 contingency table and by using receiver operating characteristic (ROC) curves. ROC analysis included calculating continuous likelihood ratios with 95% CI. Bootstrap 95% CIs were calculated to evaluate the specificity at fixed intervals of sensitivity (5000 iterations and a random number seed of 100).

Calculation of the posttest probability of borrelia infection was carried out using Bayesian rules.

The pretest probability of *Borrelia* infection equals the prevalence of disease with consideration of the symptoms and signs in the patient.

Pretest odds: pretest probability/(1-pretest probability).

Posttest odds: (pretest odds x likelihood ratio (LR)

Posttest probability: posttest odds/(posttest odds + 1)

The upper limit for the clinical normal reference interval was calculated using the robust method (CLSI C28 – A3, 2008), and the CIs were estimated using the bootstrap method (10 000 iterations, random number seed 978).

MedCalc^®^ Statistical Software version 20.116 (MedCalc Software Ltd, Ostend, Belgium; https://www.medcalc.org; 2022) was used for descriptive and analytical calculations.

## 3 Results

### 3.1 Healthy blood donors, background seropositivity

Quantitative summary comparison of the performance of the assays in 312 healthy blood donor (HBD) samples showed a proportion of antibodies of 86 (27.6% (95%CI 22.7-32.9)) using the reference method and 83 (26.6%) (95% CI 21.8-31.9)) using the index method. The difference was not significant (1%, p = 0.78) (**Table 1A**).

**Table 1:** (A, B) Antibodies to borrelia C6-peptide in serum samples from 312 healthy blood donors. (A) Summary seropositivity and seronegativity with the reference and the index methods. (B) Age and gender effect on the presence of antibodies.

A)									
Test	Result	Reference					Total		
		Pos n (%)		Neg n (%)			n (%)		
Index	Pos	75 (24)		8 (2.6)			83 (26.6)		
“	Neg	11 (3.5)		218 (69.9)			229 (73.4)		
Total n (%)		86 (27.6)		226 (72.4)			312 (100)		

B)									
		Gender n		Ref % Pos			Index % pos		
Age years		F	M	F	M	All	F	M	All
20-39		38	40	11	5	10	24	5	14
40-49		43	21	17	38	23	29	43	33
50-59		48	38	38	42	43	42	47	48
60-69		46	38	39	61	49	43	66	54
Total		175	137	27	30	28	35	39	37

The seropositivity percentage increased with age according to both methods, showing a significantly higher proportion of positives in all the older age groups compared to the 20 – 39-year-olds group (p=0.007) (**Table 1B**).

The accuracy ((True positives + True negatives)/Total) of the index method was 93.9% (95%CI 90.3 – 94.1) compared to the reference method, and the difference was significant (p<0.0001. The precision, (True positives/(True positives + False positives), was 90.4% (95% CI 82.1 - 95.7) compared to the reference method, and the difference was significant (p<0.0001). Discordant categorical results were found in 19 samples (6,09%) a significant proportion (p<0.0001).

The antibody levels were 0.385 LI (0.1 - 264.3) using the reference method and 0.66 AU (0.2 - 1597.5) using the index method.

**Figure 1:**
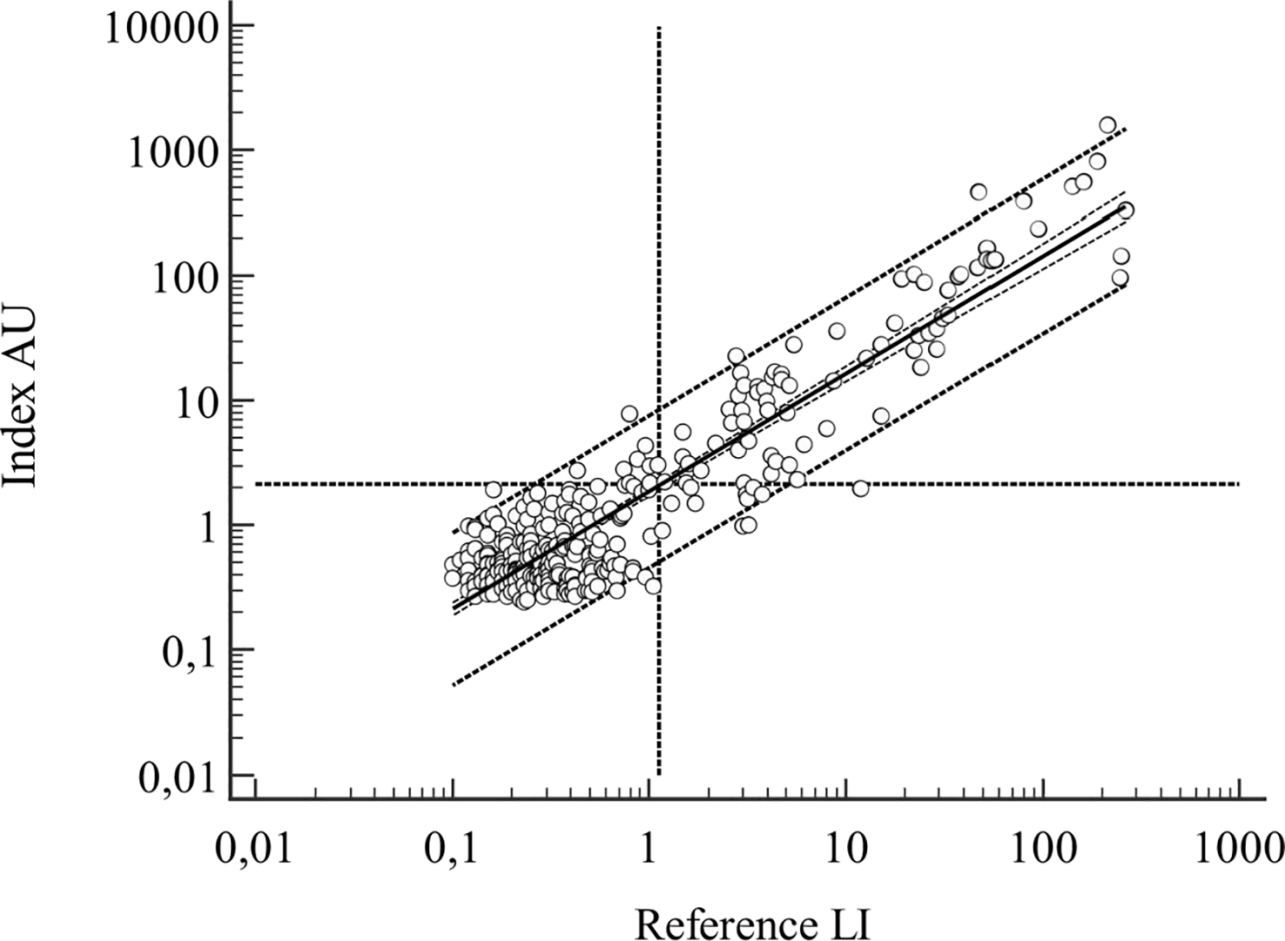
HBD samples, n=312, regression line with 95% CI and 95% prediction interval outlined, vertical and horizontal dotted lines represent the positive/negative cutoff points for the assays. Regression line: log(y) = 0.2695 + 0.942log(x), R^2 = ^0.848.

In the regression study, the relationship between the methods had a coefficient of determination R^2 = ^0.848, which was interpreted as good (**Figure 1**).

Taken together, the results in HBD demonstrated a considerable percentage of background seropositivity, seriously interfering with rule-in serodiagnostics. The observed differences between the methods were not significant in the summary data. Performance characteristics, such as accuracy and precision, were significantly different.

### 3.2 Diagnostic performance of the derivation cohort

*Borrelia* antibodies were found in 175 samples, 40.6% (95% CI 35.9 – 45.4), using the reference method, and in 172, 39.9% (95% CI 35.2 - 44.7) using the index method, the difference between the two methods (0.7%) was not significant (p=0.834).

The accuracy of the method compared to the reference method was 94.2% (95% CI 91.6 - 96.2) and the difference (5.8%) was significant (p<0.0001). The precision was 93.6% (95% CI 90.9-95.7) compared to the reference method and the difference (6.4%) was significant (p<0.0001). Discordant categorical results were found in 25 samples (5.1%) a significant proportion (p<0.0001).

**Figure 2:**
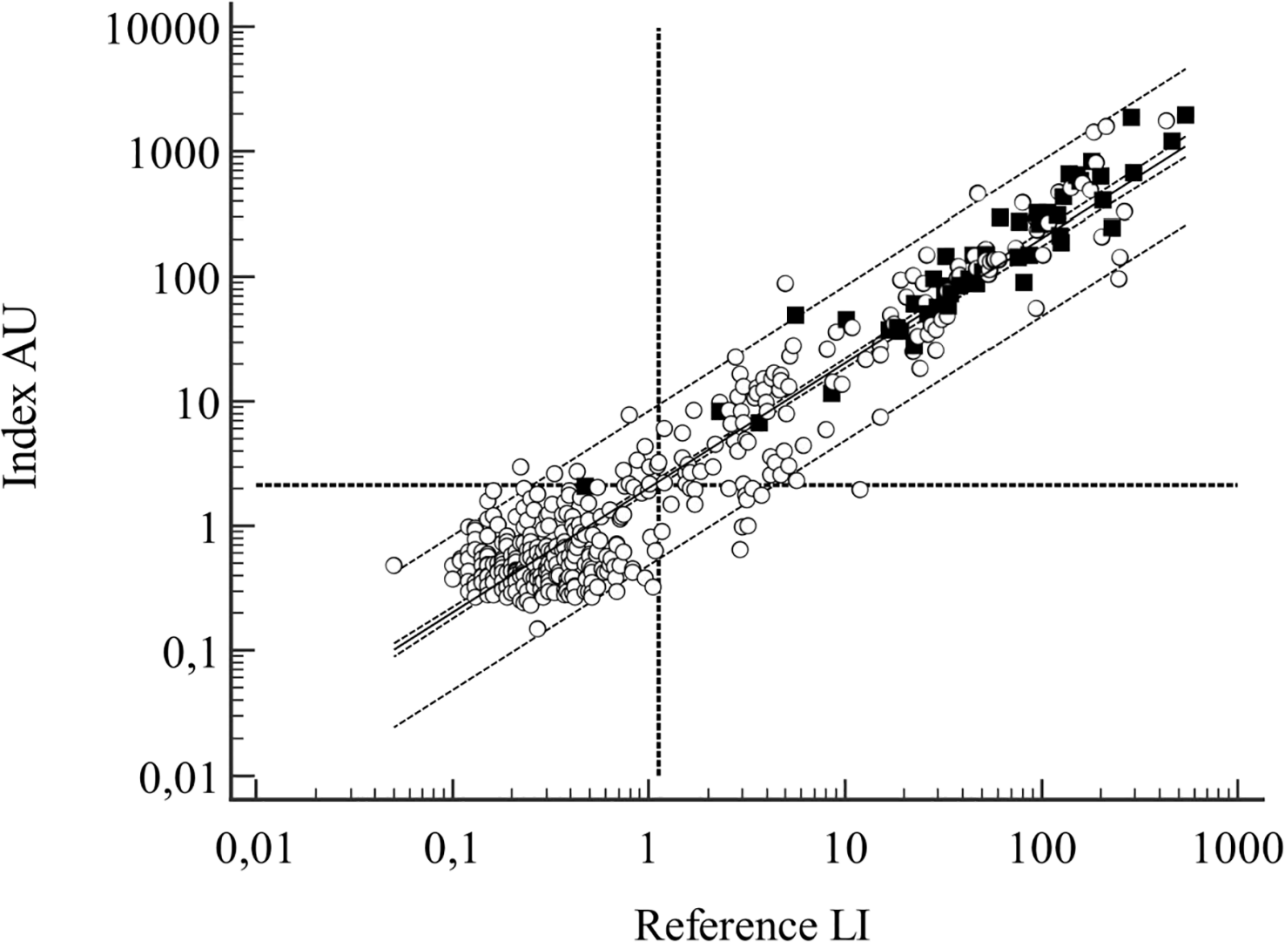
Samples from the derivation cohort, n=431. Regression line with 95% CI and 95% prediction interval outlined, vertical and horizontal dotted lines represent the positive/negative cutoff points for the assays. Filled symbols denote LB cases. Regression line: log(y) = 0,3058 + 0.9997(logx), R^2 = ^0.909.

The antibody levels were 0.52 (range 0.05-540) LI and 1.02 (range 0.15-1962) AU in the total material. The R^2^ of the regression analysis was 0.91 (**Figure 2**).

In order to assess the diagnostic performance of the antibody determinations, summary binary diagnostic and predictive parameters for the reference and the index methods were calculated.

No significant differences were observed in the binary diagnostic parameters. The sensitivity of each method was high, as technically aimed for. The false positive proportion was 73.1% for the reference test and 72.1% for the index test. The specificity reflected the seropositivity rate of the clinical population in an endemic area (**Table 2**).

**Table 2:** Comparison of binary diagnostic parameters in the derivation cohort analyzed by the reference and the index methods.

N = 431 Test Parameter	Reference (95% CI)	Index (95% CI)
Sensitivity %	97.9 (88.9 – 99.9)	100 (92.6 – 100)
Specificity %	66.6 (61.6 – 71.3)	67.6 (62.7 – 72.3)
LR +	2.9 (2.5 – 3.4)	3.1 (2.7 – 3.6)
LR -	0.03 (0.004 – 0.23)	0
PPV %	26.9 (24.10 – 29.80)	27.9 (25.10 – 30.9)
NPV %	99.6 (97.3 – 99.90)	100

CI, confidence interval; LR+ and LR-, positive and negative likelihood ratio; PPV and NPV positive and negative predictive value.

The positive predictive values (PPV) and the positive likelihood ratio (LR+) were low and unhelpful in diagnosing active *Borrelia* infection.

The negative predictive values (NPV) were nearly 100% for each method, thus useful for ruling-out *Borrelia* infection.

The summary LR- was 0.03 (95% CI 0.004-0.23) for the reference method, indicating the presence of an uncertain interval in the negative antibody results. The LR- for the index method was 0, indicating the high reliability of the negative test in ruling-out *Borrelia* infection.

**Figure 3:**
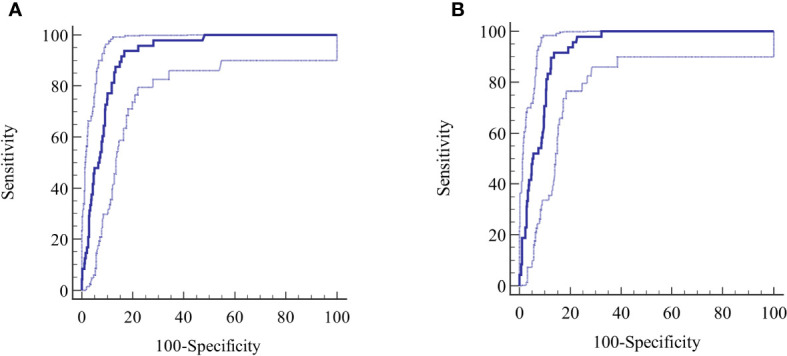
**(A, B)** Derivation cohort, ROC curves for **(A)** reference test and **(B)** index test. The calculated central ROC-line is displayed with upper and lower 95% confidence limits.

ROC-curve analysis was performed to confirm the overall diagnostic performance and to clarify the discriminatory properties of the tests over the continuum of results and the dependency of the diagnosis on the antibody level (**Figures 3A, B**).

The AUC for the reference method was 0.919 (95% CI 0.882-0.945) and 0.925 (95% CI 0.895-0.948) for the index method. The difference between the AUCs was not significant (p=0.281). Other diagnostic parameters were identical to the calculated binary values (**Table 2**).

The ROC was evaluated at several points of the curve determined by the LR values.

At an LR+ level of 5, sensitivity and specificity values of 93.8% and 81.4%, were reached at 4.75 LI for the reference method, rising to an LR+ of 7.1, a sensitivity of 50%, specificity of 93%, at 50 LI, resulting in an LB post-test probability of 0.47. Further reduction in the sensitivity was not diagnostically meaningful.

The corresponding values for the index method at an LR+ of 5 include a sensitivity of 91.7% and a specificity of 82% at 13 AU, rising to an LR+ level of 9.6, a sensitivity of 50%, and a specificity of 94.8% at 155 AU, giving a post-test LB probability 0.545.

The specificity of the tests, measured at fixed intervals of sensitivity with bootstrap 95% CI, revealed a specificity of 52.1% (95% CI 42.02-72.0) at a sensitivity of 99 for the reference test, requiring a cutoff ≥0.46 LI, considerably lower than the standard cutoff ≥1.1 LI. The same calculation for the index test gave a specificity of 67.6% (95% CI 59.8 - 77.0) at a sensitivity of 99% at standard cutoff ≥2.1 AU. The difference in specificity (-15.5%) between the methods was significant, (p=0.0003).

The lower cutoff ≥0.46 LI for the reference method, necessary to achieve a sensitivity of 99%, resulted in a reduction of the total negatives count from 256 to 198, a significant reduction of 22.7% (95% CI 17.7 - 28.3) in the count of potential true negatives, (p<0.0001). This reduction was also reflected in the LR- of the reference test. The index test at standard cutoff ≥2.1 AU detected 259 total negatives at a sensitivity of 99%.

Positive serologic results were related to active LB, but were not diagnostic for active *Borrelia* infection. In relation to active borreliosis the antibody levels differed in medians, (p<0.000001) but showed an overlapping distribution with those without borreliosis, preventing the use of antibody levels as an indicator of active disease (**Table 3**). The distribution is also evident in the regression graph (**Figure 2**).

**Table 3:** Antibody levels in samples from the derivation cohort with active borrelia infection (LB) and without active infection (Not-LB).

N=431	Sample antibody level	
Test	Not-LB median (range)	LB median (range)
Reference LI	0.43 (0.05-434.1)	51.3 (0.47-540.6)
Index AU	0.76 (0.15-1767.9)	143.6 (2.1-1961.5)

LI, Lyme index; AU, arbitrary units.

In conclusion, there were significant differences in accuracy and precision present. The index test was diagnostically not inferior to the reference test but showed a possible advantage over the reference test in terms of validity and ability to discriminate true negative levels of antibodies.

### 3.3 Diagnostic performance in the validation cohort

*Borrelia* antibodies were found in 117 samples (56.3% (95% CI 49.3 – 63.1)), using the reference method and in 114 (54.8% (95% CI 47.8 - 61.7)) using the index method, and the difference (1.3%) was not significant, (p=0.756).

The accuracy of the index versus the reference method was 92.8% (95% CI 88.4 - 95.9), a significant difference of 6.2% between the method (p<0.0014). The precision was 92.3% (95% CI 91.1 - 93.4), and there was a significant difference of 5.3% between the two methods (p=0.012). Discordant categorical results were found in 15 samples (7.3%), a significant proportion (p<0.0001).

**Figure 4:**
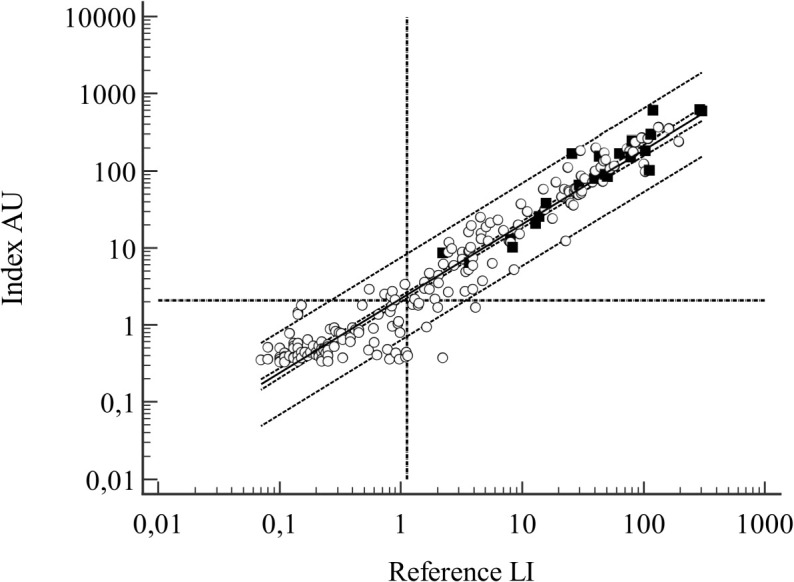
Samples from the validation cohort, n=208. Regression line with 95% CI and 95% prediction interval outlined, vertical and horizontal dotted lines represent the positive/negative cutoff points for the assays. Filled symbols denote LB cases. Regression line: log(y) = 0,3415 + 0.9997(logx), R^2 = ^0.9638.

The antibody levels were 0.52 (0.05-540) LI and 1.02 (0.15-1962) AU in the total material. The R^2^ in the regression analysis was 0.93 (**Figure 4**).

Twenty-two patients (10.6%) from the validation cohort were diagnosed with active *Borrelia* infection: 7 NB, 4 LA, 1 ACA, 4 EM and 6 early disseminated LB (LBdiss). Alternative causes were found in 176 patients and 10 seronegative individuals with unspecific symptoms were lost to follow-up (**Table 4**).

**Table 4:** Symptoms, diagnosis and type of borrelia infection, alternative diagnosis in the validation cohort.

Symptoms (N=208)	Borrelia infection (n = 22), 10.6%	Alternative causes n=176
Cognitive, neurologic 52, 25%	6 total, 12% - 4 NB/FP - 1 NB - 1 LBdiss	Dementia, vertigo, vascular, ADHD, anxiety, Parkinsons, ALS, depression, neuropathy
Skin 7, 3%	3 total, 43% - 1 EM - 1 EM+LBdiss - 1 ACA	Psoriasis, venous insufficiency, pruritus, T-lymphoma, angio- edema
Joints 35, 17%	4 total, 11% - 4 LA	Osteoarthritis, rotator cuff, trochanteritis, distorsion, gout, dysplasia, unspecific pain
Autoimmune 27, 13%	2 total, 7% - 2 LBdiss	RA, PsA, JRA, SLE, SpA, reactive arthritis, PMR, FM, sclerosing cholangitis
Unspecific 40, 19%	4 total, 10% - 2 NB, - 2 LBdiss	Constitutional symptoms, fatigue, headache, lymph-adenitis
Back 12, 6%	–	Disc degeneration, spinal stenosis, neck or lower back pain
Muscle 7, 3%	1 total, 14% - 1 LBdiss	Myalgia
Miscellaneous 28, 13%	2 total, 7% - 1 EM - 1 EM post-treatment	Otitis, Cirrhosis, hepatitis C-chronic, cancer, heart, asthma, sleep apnea, syncope, dyspnea

FP, facial palsy; ADHD, attention deficit hyperactivity disorder; RA, rheumatoid arthritis; PsA, psoriatic arthritis; JRA, juvenile rheumatoid arthritis; SLE, systemic lupus erythematosus; SpA, ankylosing spondylitis; PMR, polymyalgia rheumatica; FM, fibromyalgia.

The symptoms and clinical signs among patients in the validation cohort were multifaceted and not specific to *Borrelia* infection, except for concomitant EM.

There were no significant differences between the methods in the calculated binary diagnostic parameters (**Table 5**). The sensitivity was 100% for each assay and the false positive proportion using the reference test was 81.2% and 80.7% for the index test.

**Table 5:** Binary diagnostic parameters for the validation cohort.

N = 208 Test Parameter	Reference (95% CI)	Index (95% CI)
Sensitivity %	100 (85.2 – 100)	100 (85.2 – 100)
Specificity %	49.2 (41.8 – 56.6)	50.8 (43.4 – 58.2)
LR +	1.93 (21.7– 2.3)	2 (1.8 – 2.4)
LR -	0	0
PPV %	19.7 (17.5 – 22)	20.2 (17.9 – 22.6)
NPV %	100	100

CI, confidence interval; LR+ and LR-, positive and negative likelihood ratio; PPV and NPV positive and negative predictive value.

The specificity reflected the seropositivity rate of the clinical population in an endemic area. The PPV and the LR+ were low and had no diagnostic utility. The NPVs were 100% with each test and the summary LR- was 0 for both methods, not indicating the presence of false negative results in the samples of the validation cohort.

**Figure 5:**
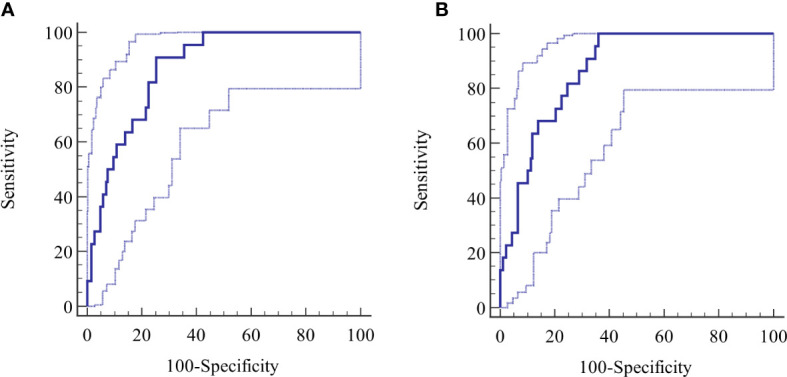
**(A, B)** Validation cohort, ROC curves for **(A)** reference test and **(B)** index test.

In ROC- curve analysis (**Figures 5A, B**) the AUC with the reference method was 0.871 (95% CI 0.80-0.921) and 0.867 (95% CI 0.80-0.917) with the index test and the difference between the AUCs was not significant (p=0.716). The diagnostic parameters were the same as the calculated binary figures.

Further evaluations of the ROCs were performed at various points of LR values. At an LR+ level of 5, the sensitivity and specificity of the reference method were 59.1% and 88.7% at 31 LI, rising to LR+ of 5.2, a sensitivity of 50%, and a specificity of 90.3% at 50 LI, resulting in an active borreliosis post-test probability of 0.49. Further reduction in sensitivity was not diagnostically meaningful.

The corresponding values at 73 AU using the index method included an LR+ of 5, a sensitivity of 63.6%, and a specificity of 87.6%. These values changed to an LR+ of 4.43, a sensitivity of 50%, and a specificity of 88.7%, at 89 AU, resulting in a post-test probability of 0.45.

The specificity of the tests, measured at fixed intervals of sensitivity with bootstrap 95% CI, revealed a specificity of 57.5% (95% CI 44.1 - 66.1) at a sensitivity of 99% for the reference test at the 2.22 LI cutoff, which was higher than the standard cutoff ≥1.1 LI. The index test had a specificity of 64% (95% CI 55.9 - 71.5) at a sensitivity of 99% at a cutoff of 6,36 AU. The difference of 6.5% in specificity was not significant (p= 0.1506).

The total negatives count at the upper limit of sensitivity of 100% was 107 with the reference method and 119 with the index method, a nonsignificant difference of 5.8% (p=0.2356). When the lower cutoff >0.46 LI for the reference method was applied, the total count of negatives reduced from 91 to 67, indicating a significant reduction of 26.4% (95% CI 17.7 - 36.7) in the count of potential true negatives (p=<0.0001). The index test at cutoff ≥2.1 AU detected a total count of 94 negatives.

Positive serologic results were related to active *Borrelia* infection but were not diagnostic for the disease. In the not-LB and LB cases, the antibody levels showed an overlapping distribution, preventing the use of antibody levels as an indicator of active disease (**Table 6**). This distribution is also evident in the regression graph (**Figure 4**).

**Table 6:** Antibody levels in samples from the validation cohort with active borrelia infection (LB) and without active infection (Not-LB).

N=208	Sample antibody level	
Test	Not-LB median (range)	LB median (range)
Reference LI	1.19 (0.07-194.1)	45.8 (2.22-102.4)
Index AU	1.88 (0.33-364.5)	95.3 (6.46-623.5)

LI, Lyme index, AU, arbitrary units.

In conclusion, the accuracy and precision of the index test related to the reference test were significantly lower in the validation cohort, implicating a difference in the performance of the tests. Diagnostically the index test performed equally to the reference test. A potential advantage of the index test over the reference test in terms of validity and ability to discriminate true negative levels of antibodies was possible, but not verified.

## 4 Discussion

Antibody tests are important in supporting the diagnostics of manifestations due to *Borrelia* infection, apart from EM. However, due to several shortcomings, the positive predictive performance of diagnostic tests based on antibodies is insufficient. This is mostly recognized by the physicians treating *Borrelia* infections and therefore, the practical use of antibody tests is based on negative test results to exclude an active infection after a symptomatic period of 2-4 weeks.

In this study, we describe the development and evaluation of a novel test for IgG1-subclass antibodies to B.b.sl. in a hyperendemic European area. The findings demonstrated a low positive predictive performance, placing special emphasis on the rule-out performance of the test.

The induction of antibodies by *Borrelia* infection is a secondary and indirect sign of a recent, ongoing, or past infection. The basic requirements for an antibody test are a high sensitivity (preferably >96%) in detecting specific antibodies, and a high specificity (ideally >95%) for antibody detection (Power et al., 2013).

These requirements were evaluated by analyzing 693 blood samples, from 2 groups, including a derivation cohort and a validation cohort. The results were compared with the figures for the well-characterized reference test. In order to simplify the discussion, the results of the accuracy, precision, and sensitivity in these groups are combined here. Antibodies to *Borrelia* were present with the reference test in 378 samples (39.7%) and in 369 samples (38.8%) with the index test. Compared to the reference test, the accuracy of the index test was 93.8% (95% CI 92.1-95.2) and the precision was 93.2% (95% CI 90.2-95.5), and these differences were significant (p<0.0001).

In terms of diagnostic metrics, and comparing antibody levels, we found a sensitivity of the index test of 91% (95% CI 87.7-93.7) compared to the reference test and specificity of 95.6% (95% CI 93.6-97.2), which significantly differed between both methods (p<0.0001).

The precision suggests that the difference in performance may be in a high (false or true) positive rate of the index test. From the sensitivity it can be deduced that a difference also exists with an increase of (false or true) negatives in the index test, indicating a symmetric distribution of discordant results.

This is supported by the 59 (6.2%), discordant categoric results, which were equally distributed to (false or true) positives 2.63% and (false or true) negatives (3.6%). The difference was not significant (p=0.062).

The findings suggest that the tests are different with respect to antibody detection. The causes of this are not explained by the present study, although this is a prerequisite for a better discriminative function.

The performance metrics for the clinician are related to those discussed above, but the diagnostic performance is of greater importance. In ideal circumstances: 1) a high sensitivity >96% is related to the presence of infection; 2) a high specificity >95%, is related to the absence of actual infection; and 3) the individual probability of being ill given the presence or absence of antibodies to borrelia can be calculated (Power et al., 2013).

In the clinical derivation and validation cohorts, the optimal sensitivity conditions are met. This is illustrated by the calculation of the diagnostic performance of both tests in the combined derivation and validation cohorts. Both tests had a mean sensitivity >96% giving a high NPV of 98 -100%, which is required for ruling out active infection. The specificity is low at 67%, mainly due to a high antibody-level background. The background antibody level is also age dependent, and the seropositive proportion is greater in the clinical population compared to HBD. Furthermore, the local seroprevalence may be unknown. Sensitivity and specificity are both strongly prevalence-sensitive parameters and as such, are not directly useful for making diagnostic decisions. The LRs are relatively prevalence insensitive and LR+ (sensitivity/1-specificity) reflects how many times the odds for disease in someone will be elevated by a positive test knowing the pre-test prevalence and using the Bayesian approach the individual post-test probability of disease can be estimated. Thus, in a population with a 20% pretest probability of borrelia infection, the odds of disease are 0.25, which multiplied by LR+ 2.5 gives post-test odds 0.625, which converted to post-test probability will be 0.385, indicating a 38.5% risk of borrelia infection.

The risk of active borrelia infection, except for EM, in patients from a high-endemicity population, is below 20% even with high alertness. With lower pre-test probabilities, the diagnostic value of a positive test is mostly negligible.

A certain level of antibodies is not a reliable sign of disease, which also is documented f or IgG1-antibodies in the present study by comparing antibody levels in HBD, in samples from patients with and without *Borrelia* infection and by ROC analysis of the LR+ distribution over positive antibody levels.

In seronegative patients, 2-4 weeks of symptomatic infection are necessary for a measurable IgG response in serum to develop. Thus, a patient with a shorter symptomatic period may be seronegative or low positive and a second antibody determination should be performed after 2-4 weeks. Therefore, there is a time window where test results may be negative or discordant.

Positive IgG antibodies to *Borrelia* C6-peptide have to be supported by epidemiology, clinical signs, and other investigations, such as Csf-studies including chemokine CXCL13 and borrelia PCR from biopsies or synovial fluid.

In this study, we found a multitude of non-specific symptoms, related to an active borrelia infection. When objective findings supporting *Borrelia* infection are not found, other than positive antibody tests, the differential diagnostic is of utmost importance, to offer the patient an explanation and a treatment for the symptoms experienced.

Negative antibody tests may be useful for excluding an active *Borrelia* infection, when certain criteria are fulfilled, including: 1) a high sensitivity >99%, 2) a low LR- <0.1, 3) knowledge of the pre-test prevalence of *Borrelia* infection in the population and 4) whether the result of the test will benefit the patient.

Each test has a sufficient level of sensitivity qualifying them as potential ruling-out tests, while diagnostically high false positive proportions definitely disqualify them as rule-in tests.

The results of the present study showed that the reference test and index test are functionally different, as demonstrated by the significant differences in accuracy, precision, and sensitivity of antibody detection.

The rule-out performance is strongly related to the LR- (1-sensitivity/specificity). This is found in the derivation cohort with the index test. The reference test classified one sample from a definite patient as seronegative. The summary LR- was then 0.03 (95% CI 0.004 - 0.23) for the reference method. Will the patient and/or the physician benefit from knowing the result? In order to achieve high sensitivity of the reference test, the cutoff level has to be reduced from 1.1 LI to 0.46 LI. The 75th percentile of negative LI results is 0.43 (95% CI 0.40-0.49). The lower limit reduces significantly the number of seronegative samples by 58, from 256 to 198, 22.7%, which may be considered as a risk interval for false negative results. The indeterminate interval for the reference method, 0.91-1.09 LI, includes 9 patients in the derivation cohort.

By applying a Bayesian approach the patient’s post-test probability of *Borrelia* infection can be calculated. In a population with a 20% pre-test probability of active *Borrelia* infection and the mean LR- 0.03 the post-test probability is 0,74%. At the upper limit of the 95% CI of LR- 0,23, the post-test probability is 5.4%. This is a considerable risk for the patients. Of 1000 patients evaluated, 7-54 may be wrongly reassured that they are not actively infected with *Borrelia*. To avoid this potential cause of seronegative *Borrelia* infections, a test unlikely to produce false negative results is advantageous. A more correct serology may also guide the patient to important differential diagnostic investigations and treatment.

## Data availability statement

The raw data supporting the conclusions of this article will be made available by the authors, without undue reservation.

## Ethics statement

The studies involving humans were approved by Local ethics committee Aland Health Services, Finnish Red Cross transfusion services. The studies were conducted in accordance with the local legislation and institutional requirements. The participants provided their written informed consent to participate in this study.

## Author contributions

DN conceived the novel test, performed statistical analyses, and wrote the manuscript. MN and CN were responsible for clinical workup and treatment. SO and NC-B performed the laboratory part, and S-AC performed data registration. All authors were involved in the writing and approval of the manuscript.
